# Supplementary material for: Host IP3R channels are dispensable for rotavirus Ca2+ signaling but critical for intercellular Ca2+ waves that prime uninfected cells for rapid virus spread
Source: mBio. 2023 Dec 19;15(1):e02145-23. doi: 10.1128/mbio.02145-23 (PMC10790754; doi:10.1128/mbio.02145-23)
Supplement: Supplemental movie captions — Legends for Movies S1-S7. [file mbio.02145-23-s0001.docx]

**Supplemental Movie Legends**

**Movie 1.** MA104-GCaMP6s cells were mock (left) or infected with SA11-mRuby (right) and live time-lapse imaging was performed. GCaMP6s reports cytoplasmic Ca^2+^ as changes in fluorescence intensity (green) and mRuby fluorescence (red) reports rotavirus protein synthesis from gene 7. GCaMP6s and mRuby channels were imaged with a 1-minute and 10-minute interval between image captures, respectively. Note that imbedded timer displays time from the beginning of the acquisition not the time post-infection.

**Movie 2.** HEK293-GCaMP6s cells (left) or HEK293-GCaMP6s-IP_3_R-TKO cells (right) were infected with SA11-mRuby and live time-lapse imaging was performed. GCaMP6s reports cytoplasmic Ca^2+^ as changes in fluorescence intensity (green) and mRuby fluorescence (red) reports rotavirus protein synthesis from gene 7. GCaMP6s and mRuby channels were imaged with a 1-minute and 10-minute interval between image captures, respectively. Note that imbedded timer displays time from the beginning of the acquisition not the time post-infection.

**Movie 3.** MA104-GCaMP6s cells (left, top and bottom) or MA104-GCaMP6s-IP3R-TKO cells (right, top and bottom) were treated with 50 µM ADP (top) or 0.75 µM AC5541, a protease-activated receptor 2 (PAR2) agonist during live time-lapse imaging GCaMP6s fluorescence for changes in cytosolic Ca^2+^. Images for acquired with a 1-second interval between captures. Note that imbedded timer displays time from the beginning of the acquisition not the time post-infection.

**Movie 4.** MA104-GCaMP6s cells (left) or MA104-GCaMP6s-IP3R-TKO cells (right) were infected with SA11-mRuby (right) and live time-lapse imaging was performed. GCaMP6s reports cytoplasmic Ca^2+^ as changes in fluorescence intensity (green) and mRuby fluorescence (red) reports rotavirus protein synthesis from gene 7. GCaMP6s and mRuby channels were imaged with a 1-minute and 10-minute interval between image captures, respectively. Note that imbedded timer displays time from the beginning of the acquisition not the time post-infection.

**Movie 5.** MA104-GCaMP6s cells (left) or MA104-GCaMP6s-IP3R-TKO cells (right) were infected with SA11-mRuby (right) and maintained in FluoroBrite-Plus media with 1 µg/mL trypsin (see Materials and Methods section) during live time-lapse imaging. GCaMP6s reports cytoplasmic Ca^2+^ as changes in fluorescence intensity (green) and mRuby fluorescence (red) reports rotavirus protein synthesis from gene 7. GCaMP6s and mRuby channels were imaged with a 1-minute and 10-minute interval between image captures, respectively. Note that imbedded timer displays time from the beginning of the acquisition not the time post-infection.

**Movie 6.** LLC-MK2-GCaMP6s cells (left) or LLC-MK2-GCaMP6s+P2Y1 cells (right) were treated with 25 nM ADP during live time-lapse imaging GCaMP6s fluorescence for changes in cytosolic Ca^2+^. Images for acquired with a 1-second interval between captures. Note that imbedded timer displays time from the beginning of the acquisition not the time post-infection.

**Movie 7.** LLC-MK2-GCaMP6s cells (top) or LLC-MK2-GCaMP6s+P2Y1 cells (bottom) were mock inoculated (left, top and bottom) or infected with SA11-mRuby (right, top and bottom) and live time-lapse imaging was performed. GCaMP6s reports cytoplasmic Ca^2+^ as changes in fluorescence intensity (green) and mRuby fluorescence (red) reports rotavirus protein synthesis from gene 7. GCaMP6s and mRuby channels were imaged with a 1-minute and 10-minute interval between image captures, respectively. Note that imbedded timer displays time from the beginning of the acquisition not the time post-infection.
